# Influence of ocean acidification on plankton community structure during a winter-to-summer succession: An imaging approach indicates that copepods can benefit from elevated CO_2_ via indirect food web effects

**DOI:** 10.1371/journal.pone.0169737

**Published:** 2017-02-08

**Authors:** Jan Taucher, Mathias Haunost, Tim Boxhammer, Lennart T. Bach, María Algueró-Muñiz, Ulf Riebesell

**Affiliations:** 1 GEOMAR Helmholtz Centre for Ocean Research, Kiel, Germany; 2 Alfred-Wegener-Institut Helmholtz-Zentrum for Polar and Marine Research, Biological Institute, Helgoland, Germany; Stazione Zoologica Anton Dohrn, ITALY

## Abstract

Plankton communities play a key role in the marine food web and are expected to be highly sensitive to ongoing environmental change. Oceanic uptake of anthropogenic carbon dioxide (CO_2_) causes pronounced shifts in marine carbonate chemistry and a decrease in seawater pH. These changes–summarized by the term ocean acidification (OA)–can significantly affect the physiology of planktonic organisms. However, studies on the response of entire plankton communities to OA, which also include indirect effects via food-web interactions, are still relatively rare. Thus, it is presently unclear how OA could affect the functioning of entire ecosystems and biogeochemical element cycles. In this study, we report from a long-term *in situ* mesocosm experiment, where we investigated the response of natural plankton communities in temperate waters (Gullmarfjord, Sweden) to elevated CO_2_ concentrations and OA as expected for the end of the century (~760 μatm pCO_2_). Based on a plankton-imaging approach, we examined size structure, community composition and food web characteristics of the whole plankton assemblage, ranging from picoplankton to mesozooplankton, during an entire winter-to-summer succession. The plankton imaging system revealed pronounced temporal changes in the size structure of the copepod community over the course of the plankton bloom. The observed shift towards smaller individuals resulted in an overall decrease of copepod biomass by 25%, despite increasing numerical abundances. Furthermore, we observed distinct effects of elevated CO_2_ on biomass and size structure of the entire plankton community. Notably, the biomass of copepods, dominated by *Pseudocalanus acuspes*, displayed a tendency towards elevated biomass by up to 30–40% under simulated ocean acidification. This effect was significant for certain copepod size classes and was most likely driven by CO_2_-stimulated responses of primary producers and a complex interplay of trophic interactions that allowed this CO_2_ effect to propagate up the food web. Such OA-induced shifts in plankton community structure could have far-reaching consequences for food-web interactions, biomass transfer to higher trophic levels and biogeochemical cycling of marine ecosystems.

## 1 Introduction

Over the past few centuries, anthropogenic emissions of carbon dioxide (CO_2_) resulted in an increase of atmospheric concentrations from average preindustrial levels of approximately 280 to more than 400 ppmv (parts per million volume) at present [1]. About one third of this carbon is currently taken up by the world oceans [2, 3], leading to a decrease in pH and pronounced shifts in seawater carbonate chemistry that occur at a pace unprecedented in recent geological history [4, 5]. This process, which is commonly referred to as “ocean acidification” (OA), is expected to have substantial consequences for marine ecosystems [6, 7]. Over the last few years, an increasing number of studies have investigated effects of OA on marine organisms, revealing that the response of different organism groups to increasing CO_2_ is highly variable [8–13]. For zooplankton, research efforts focused mainly on copepods, and mostly suggest a rather low sensitivity to ocean acidification [14–16], although recent evidence indicates that certain life stages might be more sensitive [17, 18]. However, most of these studies were conducted with single species and / or artificial predator-prey combinations, making it difficult to estimate how observed effects might eventually translate to the responses on the community or ecosystem level in the real ocean.

Plankton communities form the base of the pelagic food web and provide many important ecosystem services such as productivity, sustenance of fish stocks, or carbon uptake. All these services are ultimately controlled by the interplay between community composition and food web structure at the lower trophic levels of marine plankton [19, 20]. For instance, zooplankton-phytoplankton coupling plays a key role in controlling the development of large-scale plankton blooms [21, 22] and influences the magnitude and efficiency of the biological pump [23, 24]. Because top-down control by predators can have a strong impact on productivity, biodiversity and ecosystem functioning [20, 25], addressing the question of “who eats whom?” is the key to an improved mechanistic understanding of marine ecosystems. However, marine food webs are highly complex networks of interacting organisms that span several orders of magnitude in body size [26]. Furthermore, since reproductive rates of planktonic organisms are relatively high, with a time scale of days to weeks, plankton communities react rapidly to changes in environmental conditions and usually display substantial fluctuations in population sizes and community composition on short time scales [27, 28]. Consequently, it remains one of the major challenges in biological oceanography to find general rules that explain and predict the trophic structure and biogeochemical functioning of marine ecosystems and how underlying ecological processes are affected by environmental drivers, particularly in the context of ongoing climate change and ocean acidification.

One promising approach to investigate the food web structure of entire ecological communities and to tackle these complex questions is to utilize information on the size distribution of plankton. The body size of an organism is commonly considered a key property to characterize its physiology and ecology. The rates of many important biological processes have been shown to scale systematically with body mass (or any other measure of body size), ranging from small microbes up to the largest aquatic organisms [26, 29, 30]. Such size-dependent relationships have been described e.g. for growth, fecundity, and basal metabolic rate [29–31]. Furthermore, body size has a major influence on the position of an organism in the marine food web and is thus commonly considered a “master” trait in shaping the structure and functioning of marine ecosystems [32–34]. Most predators typically feed on prey in a certain size range smaller than themselves, as they can neither ingest organisms that are too large nor feed efficiently on organisms that are too small [35, 36]. Accordingly, prey size spectra of dominant predator species in a given system can heavily influence food web structure and associated flow of biomass and energy [37]. Sheldon et al. [38] for the first time described large-scale patterns in the size distribution of particles in different oceanic regions. Their concept of the “size spectrum” comprises several mathematical representations of the general relationship between the abundance of organisms and their body size. This inverse relationship is usually linear on a logarithmic scale, with steeper slopes indicating a higher proportion of smaller organisms and shallower slopes indicating a higher proportion of larger organisms [39]. Based on this concept of the marine size spectrum, an entire sub-branch of marine ecology has emerged, investigating the mechanisms behind body size—abundance relationships in aquatic ecosystems [40–42]. Interestingly, power-law relationships of size spectra are very similar for very different organism size classes, ranging from phytoplankton up to large fish species [43–45]. Therefore, such size spectra can be used to compare ecosystems in terms of food web structure and energy fluxes (e.g. transfer efficiency to larger organisms), regardless of species composition [39, 46]. For instance, variations in the slope of the size spectrum can be linked to differences in the efficiency of biomass transfer to higher trophic levels [41, 47]. Similarly, anomalies in the shape of the log-transformed plankton size distribution that become visible as “waves” that deviate from the linear pattern, can indicate imbalances of growth vs. loss of different plankton populations, e.g. seasonal dynamics during a bloom or developing zooplankton cohorts that travel along the size spectrum [42, 48].

Studying the food web structure in plankton communities is particularly challenging due to its pronounced temporal variability. In order to capture and understand short-term phenomena such as plankton blooms and associated changes in species succession and trophic linkages, scientists would need datasets on plankton community composition and size distribution at a high temporal resolution (days or weeks) and over extended periods of time (several weeks to months). However, traditional methods such as microscopy are very labor-intensive and time-consuming and usually cannot provide the required temporal resolution over longer periods. This is the reason why such urgently needed datasets are very rare.

Over the past two decades, image-based systems have emerged as a valuable tool for acquiring data on plankton community composition and food web structure at much higher spatial and temporal resolution than traditional microscopic methods [49, 50]. One of the major advantages of these methods is that classification of imaged organisms can be carried out (semi)automatically by specially-designed software packages, thereby allowing for a much higher data throughput. Computer-based data analysis routinely includes measurements of size and biomass for each detected object, which can be used for calculations of particle size distribution. An increasing number of studies have demonstrated the potential of these image-based methods for revealing spatial and temporal patterns of plankton communities and food-web structure [51, 52]. This includes investigations of longer-term variability of plankton size distribution, e.g. on seasonal to annual time scales [43, 48]. However, only very few studies so far have monitored a plankton succession at sufficiently high temporal resolution to unravel shifts in community composition and food-web structure [28]. To the best of our knowledge, such an endeavor has been not yet been undertaken for a spring plankton bloom as typical for temperate and subpolar regions. In mid-latitudes, these blooms typically occur with the shoaling of the mixed layer depth in early spring, often covering large spatial scales and acting as the single biggest seasonal driver of variation in marine plankton communities and size spectra [53, 54]. During such events, phytoplankton biomass often increases by a factor of 5 to 10 within several days to few weeks [21, 55]. This phytoplankton bloom is usually followed by an increase in abundance of zooplankton, which in turn provides a critical link to higher trophic levels such as fish larvae [56, 57].

A specific aim of this study was to assess the effect of ocean acidification on plankton community structure and biogeochemical cycling during a natural spring bloom and winter-to-summer succession [58]. To address this question, we used an imaging-based approach to obtain data on size distribution and taxonomic composition of the plankton communities during an *in situ* mesocosm experiment. Pelagic *in situ* mesocosm experiments with natural plankton communities and several trophic levels have emerged as a suitable tool to study entire marine food webs, as they allow for investigating species interactions and competition in a close-to-natural environment [59, 60]. One of the major advantages of such mesocosms is that they are closed systems, allowing the same water body to be sampled over an extended period of time. This permits observing a specific plankton community without processes such as advection or migration, which usually make it difficult to track ecological populations over time. We followed the development of the plankton communities during an entire spring bloom and winter-to-summer succession, with a particular focus on potential ecological responses to simulated ocean acidification.

## 2 Methods

### 2.1 Mesocosm experiment

In the following, we will give a brief overview of the technical details of the mesocosm infrastructure that we used in the present study. For a comprehensive description of experimental design and technical details, please refer to the study by Bach et al. [58]. Briefly, the experimental setup consisted of ten pelagic mesocosms [59], which we deployed in the Gullmar Fjord (Sweden) in January 2013. The mesocosms extended to a depth of 19 m, thereby enclosing on average 50 m^3^ of the natural water column. The ten mesocosms were separated into two treatments, an untreated control (mesocosms M1, M3, M5, M9, M10) and an “ocean acidification” treatment (mesocosms M2, M4, M6, M7, M8) that simulated carbonate chemistry conditions in seawater that are likely to be expected for the end of the century (~760 μatm average pCO_2_). Target conditions for carbonate chemistry were reached and maintained by adding known amounts of CO_2_-saturated seawater to the mesocosms several times during the study. We followed the development of the enclosed plankton communities for 113 days, thereby covering the spring bloom and winter-to-summer succession. Note that patchily distributed nekton and large zooplankton like fish larvae or jellyfish were excluded from the enclosed water bodies by using nets of 1 mm mesh size. However, herring and sea urchin larvae were introduced in known quantities in the middle of the experiment, to investigate the effects of ocean acidification on higher trophic levels. For details on experimental setup and addition of organism larvae see studies by Bach et al. [58] and Sswat et al. (in prep.) and Dupont et al. (in prep.).

### 2.2 Phytoplankton: Flow cytometry and size estimation

Water column samples were collected every 2 days with “integrating water samplers” (IWS, Hydrobios) that sample a total volume of 5 L evenly throughout the water column of the mesocosms (0–17 m depth). Flow cytometry samples for the phytoplankton size range were measured within three hours after sampling with an Accuri C6 flow cytometer (for details see Bach et al., 2016). While flow cytometers do not directly measure particle size, this can be derived from light scattering properties of detected particles. Different approaches exist for doing this, and most of them are only valid for the respective instrument and calibrations. In order to determine particle size from flow cytometry data in this study, we developed an equation to convert forward scatter (FSC) to equivalent spherical diameter (ESD) of the detected particles. In a first step, we size-fractionated the samples with a variety of polycarbonate filters (0.2, 0.8, 2, 3, 5, 8 μm) following Veldhuis and Kraay [61] on several days throughout the experiment and ran them as regular samples on the flow cytometer. Increasing the nominal pore size during filtration of the samples resulted in larger particles to occur in the samples and to be detected by the flow cytometry. For each incremental increase in pore size, a class of particles with higher FSC values was detected, which could then directly be linked to the expected particle diameter based on filter pore size. Thereby we derived an FSC-to-ESD relationship of:

$$ESD=0.0064\times {FSC}^{0.5262}$$ (1)

with ESD in [μm] and FSC being a dimensionless number of forward scatter area. The obtained size distribution and temporal development of different size classes of phytoplankton are in good agreement with the data shown by Bach et al. (in prep.) who set gates for various phytoplankton groups that were based on red and orange fluorescence signal in addition to FSC.

Following the above procedure, we obtained counts for particles in the size range of approximately 0.5 to 60 μm. Based on this data, we distinguish between pico- (< 2 μm), nano- (2–20 μm) and microphytoplankton (> 20 μm). Besides phytoplankton, the acquired data also includes most heterotrophic and mixotrophic microzooplankton in this size range (e.g. ciliates), as they usually contain some chlorophyll from either ingested prey or (klepto-) chloroplasts and are therefore also detected by the fluorescence trigger. It should be kept in mind that the presented numbers are no direct measurements of cell size, and that applied FSC-to-ESD conversions might yield some deviations from “real” cell size, especially when particles deviate markedly from spherical shape. In particular at small cell diameters, rather small changes in cell diameter can have a relatively large influence on computed cell volume and biomass. Furthermore, it should be noted that the flow cytometer was set to a fluorescence trigger. Therefore, small non-fluorescing particles (e.g. bacteria, viruses, small detritus) were not detected and thus excluded from analysis. However, since particle abundances in the size range of interest (> 0.5 μm) are usually dominated by phytoplankton cells, we are confident that the contribution of non-fluorescing particles to total abundances and biomass in the investigated size range is minor and does not affect the main conclusions of our study.

### 2.3 Zooplankton: Sampling, image acquisition and processing

Samples for mesozooplankton and other larger particles were collected with a zooplankton net (Apstein, 55 μm mesh size) with 17 cm diameter. The net was pulled upwards through the water column in the mesocosms from a depth of 17 m, thus sampling a total volume of 385 L usually every 8 days [58]. Note that the sampling frequency of zooplankton net tows was restricted to every 8^th^ day in order to avoid the removal of too much zooplankton biomass, which would potentially have affected predator-prey coupling in the mesocosms.

Each net sample was collected in a plastic cup and filled up to 500 ml with filtered seawater. The majority of this sample was used for microscopic counts and analysis of taxonomic composition (see study by Algueró-Muñiz et al., in prep). 20 ml subsamples, corresponding to 4% of the total sample from the net tow (4% of 385 L = 15.4 L) were taken for image-based analysis of zooplankton community composition and size structure following the “ZooScan” method [62]. For that, the subsample was carefully poured onto glass plates (180 x 215 mm) for image acquisition. Before scanning, we manually separated and distributed objects on the glass plates in order to avoid touching or overlapping of objects on the images, which can severely impair subsequent classification of organisms and data analysis [63]. Digital images were acquired with an EPSON V750 Pro flatbed scanner at a resolution of 2400 dpi, corresponding to a pixel size of 10.6 μm. To avoid movement of organisms during image acquisition (motion blur), the subsample was fixed with a small amount of ethanol. We scanned the entire subsample without any further size-fractionation. This ensures a minimal alteration and loss of organisms and particles that can occur during size-fractionation with sieves.

Raw images were analyzed with the imaging software “ZooProcess” [62]. The software consists of a macro package in Java language for ImageJ [64]. For extraction of single target objects from the raw image, the software segments regions of interests from the raw image based on a specific grey level threshold and saves each target image into a separate file with unique ID. Furthermore, the software calculates a large number of variables for object characterization, including area, several measures of body size, biovolume and other geometric parameters.

**Fig 1:**
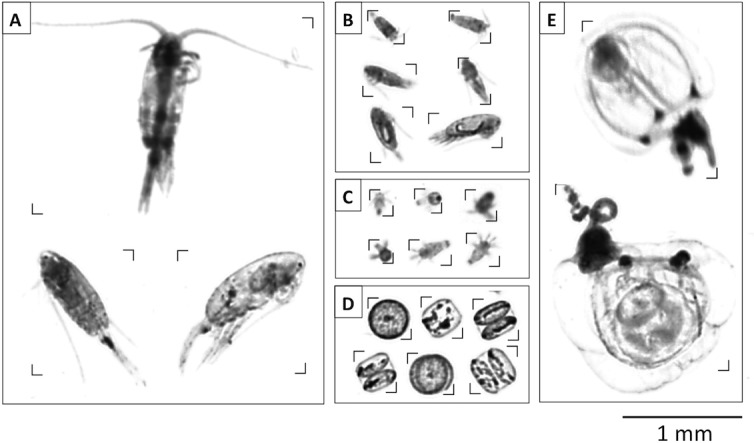
Images of organism classes (obtained with the ZooScan method) that made up a significant portion of overall biomass. A: Large copepods (*Pseudocalanus acuspes*), B: small copepods (likely copepodites), C: copepod nauplii, D: *Coscinodiscus* sp. cells, E: Hydromedusae. Scale bar is identical for all panels.

In a next step, we established a learning set for automatic classification of imaged objects. As a certain amount of pixels is required to reliably identify zooplankton specimen, the minimum size of classified organisms was restricted to ~150 μm. We created 15 categories and manually sorted a sufficiently large number of training images into the target categories (~100–200 images per category). The most important categories included copepods (mainly *Pseudocalanus acuspes*), nauplii, phytoplankton cells of the large diatom *Coscinodiscus* sp., and gelatinous zooplankton (mainly hydrozoa such as *Hybocodon prolifer* and *Aglantha digitale*) (see Fig 1). It should be noted that distinguishing between copepod life stages was not possible based on image data, due to the relatively small size of most organisms. Therefore, we grouped copepods according to their body size (based on equivalent spherical diameter, ESD) into small (*S*, < 600 μm), medium (*M*, 600–1000 μm) and large (*L*, > 1000 μm) individuals. Comparison with stereomicroscopy suggests that *M* and *L* correspond to adult copepods of different size, whereas *S* consists of different copepodite stages.

Furthermore, we established several object classes for non-living material, such as marine snow or fibers, which were then pooled into a category for “other biomass”. Touching objects or bubbles on the scanning tray were classified as “disturbances” and subtracted from the data before determining plankton abundances, biomass, and particle size spectra [63].

Automatic classification of objects from all raw images was performed using the “Plankton Identifier” software package, which uses supervised learning algorithms implemented in TANAGRA, a freely available software package for statistical analysis [62, 65]. Briefly, the software automatically sorts all (unknown) objects by applying decision mechanisms that associate object features extracted by the image analysis (e.g. grey level distributions, geometric measures) with object classifications of the human-made learning set. For more detailed descriptions of machine vision and supervised learning see e.g. Sigaud and Wilson [66] or Geurts et al. [67]. We used the “random forest” algorithm for classification, as it has been widely used in similar studies and usually yields the best results [62, 68]. Results from the automated classification were then validated manually, i.e. objects that were sorted in to the wrong category were assigned the correct category affiliation in order to achieve the highest possible accuracy for further analysis of data from the various identified plankton groups.

### 2.4 Measures of particle size distribution

There are several approaches to compute particle size spectra that all provide a useful way to describe the relationship between abundance or biomass of particles and their respective size [46, 69]. Particle size, in turn, can be expressed by various measures such as body length, equivalent spherical diameter (ESD) or biovolume.

The most commonly used particle size spectrum (“PSS” also called “number size spectrum” or “normalized abundance spectrum”) is calculated by counting the number of particles in logarithmically spaced size classes (*s*, usually based on ESD), where the abundance for each size class is divided by the linear width of the size class (Δ*s*) [69, 70]:

$$PSS(s)=\frac{abundance\;in\;size\;interval\;\Delta s}{width\;of\;size\;interval\;\Delta s}$$ (2)

The normalization procedure is carried out to correct for distortions in the underlying abundance distribution due to logarithmic binning, in which size classes increase in width proportionally with body size. The resulting particle size spectrum (PSS) represents the concentration of particles per unit size interval (*s*) in the unit [# L^-1^ mm^-1^]. Such particle size spectra usually have linear slopes of -2. However, it should be noted that near-linearity of the particle size spectrum can be deceiving, because the large range of 10–12 orders of magnitude on both x- and y-axis tends to mask changes that are small relative to this wide range, but that are important in mass distribution and ecological processes. The reason is that small deviations from the straight line in a log-log plot (i.e. the PSS) constitute large variations in terms of absolute biomass. Therefore, we also computed a weighted biomass distribution (“WBS”, also known as “weighted differential biomass distribution” [69, 71]) to visualize the actual distribution of biomass over the size continuum:

WBS(s)=PSS(s)×biomass(s)×widthofsizeintervalΔs (3)

Biomass for each object was derived from image-based biovolume, assuming a constant density of organic matter based on literature (1.060 g cm^-3^ [71–73]). Thus, estimated biomass corresponds to wet weight of organisms and particles. The WBS allows us to see the size structure at small sizes without giving it undue weight. Basically, this corresponds to the absolute amount of biomass in the respective (log-spaced) size classes, given in the unit [mg L^-1^]. The WBS is usually much more variable than the PSS without a clear negative slope and often displays several distinct peaks in different size classes [46, 69].

### 2.5 Selection of days for quantitative comparison and statistical analysis of CO_2_ effects

In order to facilitate quantitative data analysis and visualization of particle size spectra, we chose to compare two days in the experiment in more detail, which represent the most important ecological stages during the study period. These are day t1 (initial conditions, late winter) and t57 (peak of plankton bloom, i.e. during period of highest total biomass [58]).

Average values are given by the arithmetic mean of replicate mesocosms (n = 5) and its standard error (SE). We conducted independent two-sample t-tests to assess statistical significance (threshold *p*-value = 0.05) of observed effects of simulate ocean acidification on selected parameters, e.g. biomass of plankton groups or particle size distributions. Requirements for homogeneity of variances and normal distribution were assessed beforehand and accounted for in the t-test.

## 3 Results

In this study, we focus on the size distribution and composition of the plankton community, with a particular emphasis on the data obtained from the plankton-imaging platform. Other aspects of the mesocosm experiment are investigated in more detail in other studies in this PLOS collection (see study by Bach et al. [58] for an overview).

### 3.1 Development of size distribution, biomass and food-web structure during the experiment

The image-based analysis of particles revealed that there were only few organism groups that constituted the largest portion of biomass in the system (Fig 1). Mesozooplankton was dominated by copepods, with the calanoid species *Pseudocalanus acuspes* alone accounting for ~97% of detected organisms (see Algueró-Muñiz et al., in prep.). Later in the experiment (from t33 on), there was also some occurrence of hydrozoa (mainly *Hybocodon prolifer*), however, not in all of the mesocosms and in rather low abundances. Other mesozooplankton species and functional groups such as chaetognaths and appendicularia were also present, but contributed only very little to overall abundances (see Algueró-Muñiz et al., in prep.) and biomass. A notable feature was the pronounced bloom of the large diatom *Coscinodiscus* sp., which reached cell diameters of 200 to >400 μm and thereby covered the same size range as parts of the copepod community (nauplii, small copepods; Fig 1).

**Fig 2:**
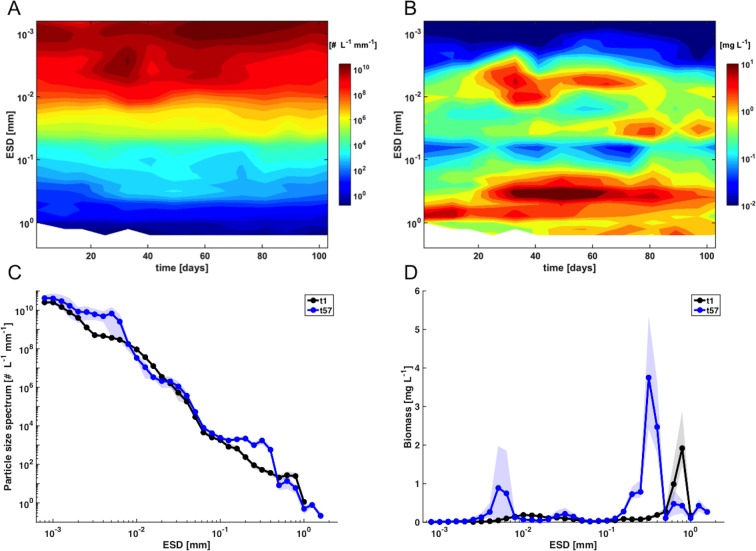
Temporal development of size distribution in the control mesocosms (average) over the course of the experiment. (A): normalized particle size spectrum, (B) weighted biomass spectrum. Note that particle diameter (ESD), as well as abundance and biomass are displayed on a log10-scale. (C) and (D): same as in (A) and (B), respectively, but focusing on t1 and t57. Shaded area denotes range of replicate mesocosms. Note that biomass in (D) is shown on a semi-log scale (i.e. linear y-axis) and not on a log-log-scale as in (B).

**Fig 3:**
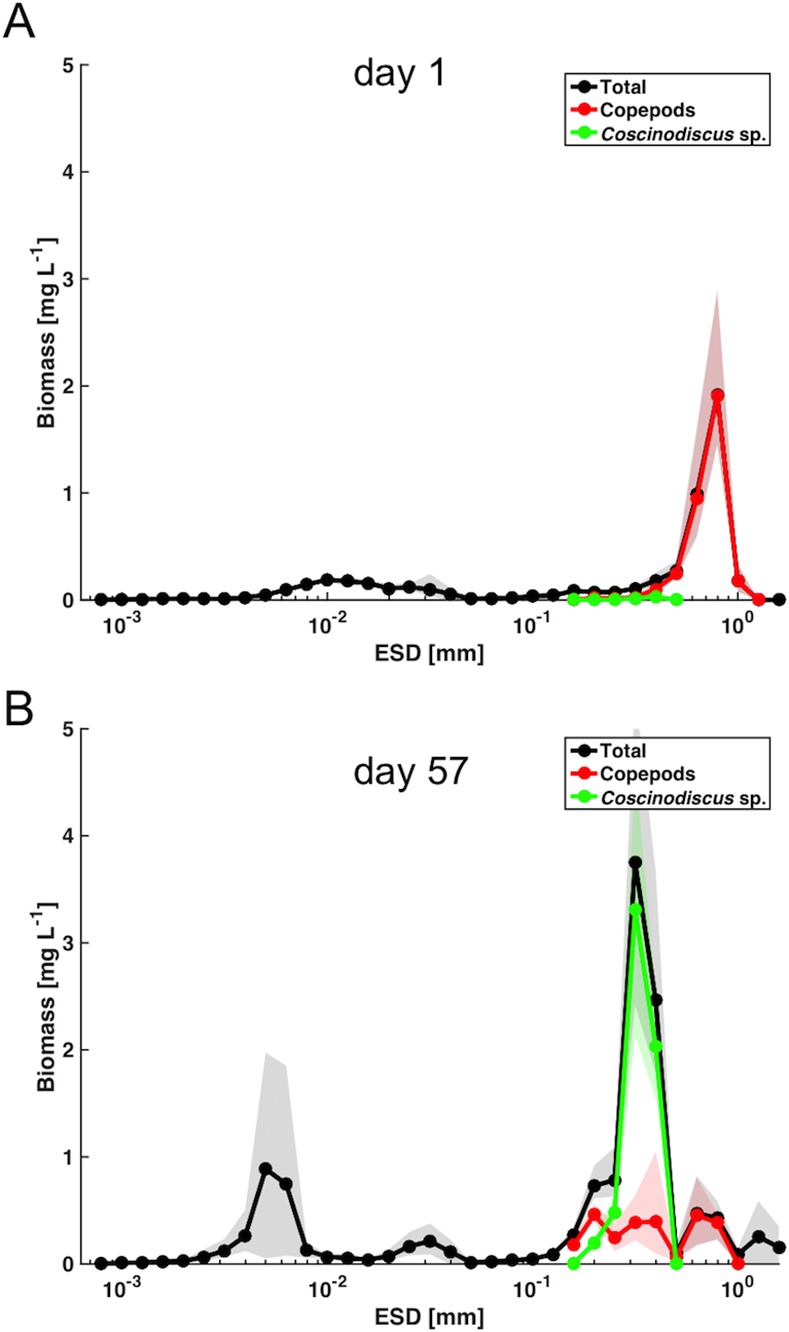
Average contribution of copepods and *Coscinodiscus* sp. to the biomass size distribution in the control mesocosms. (A) initial conditions (t1). (B) during period of maximum biomass (t57). Shaded area denotes range of replicate mesocosms.

The mesocosm experiment started in late winter with relatively low biomass concentrations (~5 mg L^-1^) and a uniform size distribution over most of the particle size spectrum on day t1 (Fig 2A and 2C). Thus, the particle size spectrum on t1 was close to linear, with a slope of -3.29 (R^2^ = 0.96). A notable feature, which becomes much more prominent in the weighted biomass spectrum, is the disproportionally large contribution of larger particles between 600–1000 μm size to overall biomass (Fig 2B and 2D). This size range accounted for >60% of total biomass and was dominated by relatively large copepods of (up to 1 mm ESD) and to a lesser extent by phytoplankton in the size range of 5–40 μm (Fig 3, Table 1).

**Table 1:** Temporal changes in plankton community size structure. Average biomass of *Coscinodiscus* sp. and different size classes of copepods in the control mesocosms on t1 and t57 (± standard error, SE).

Biomass in mesocosms (average of control treatment) [mg L^-1^]	t1			t57		
	Mean		SE	Mean		SE
Total biomass	5.26	±	0.47	12.74	±	2.01
*Coscinodiscus* sp.	0.05	±	0.01	6.02	±	0.67
Copepod total	3.43	±	0.47	2.58	±	0.40
Copepod *L* (> 1mm)	0.18	±	0.04	0.00	±	0.00
Copepod *M* (600–1000 μm)	2.85	±	0.40	0.84	±	0.13
Copepod *S* (<600 μm) & nauplii	0.40	±	0.04	1.73	±	0.32

Both the absolute amount of biomass and the relative contribution of the different plankton groups changed fundamentally over the course of the study. About two weeks after the beginning of the experiment, the development of a phytoplankton bloom became visible in a decrease of inorganic nutrients and a concomitant increase in chlorophyll *a* [58]. The emergence of this bloom was reflected in a substantial increase of total biomass, which reached concentrations that were elevated by a factor of 3 to 4 during the bloom peak, compared to initial conditions (Fig 2B). The biomass increase was mostly driven by two distinct populations in the size spectrum, being visible as “waves” in the size range of approximately 2–15 and 200–500 μm (Fig 2B).

These size classes correspond to a nanophytoplankton population, mainly consisting of small diatoms such as *Arcocellulus sp*. and *Minidiscus sp*., as well as the large diatom *Coscinodiscus* sp. [58]. In terms of biomass, the latter strongly dominated the entire system, accounting for 40–50% of total biomass on t57, whereas nanophytoplankton contributed around 20% (Fig 3, Table 1). In terms of temporal dynamics, nanophytoplankton displayed two distinct peaks around t33 and t57, whereas larger particles had a single peak around t57, but were constantly elevated for an extended period between t40 and t65 (Fig 2A and 2B). Notably, the second nanophytoplankton bloom peak around t57 displayed a narrower size range (~4–8 μm) than the first maximum on t33 (~2–15 μm). Furthermore, there was a notable increase in the picoplankton size range (< 2 μm) peaking between ~t57 and t65 with abundances increased by a factor of 3 to 4 compared to initial conditions (Fig 2A and 2C).

The phytoplankton bloom resulted in a pronounced increase in copepod abundance from 20 ind. L^-1^ on t1 to 175 ind. L^-1^ on t57. While most of this numerical increase was driven by nauplii, this development was also visible in copopods (*S* and *M*), which more than doubled from 15 to 35 ind L^-1^ between t1 and t57. These numbers agree well with abundance estimates from microscopy (Alguero-Muniz et al., in prep.).

**Fig 4:**
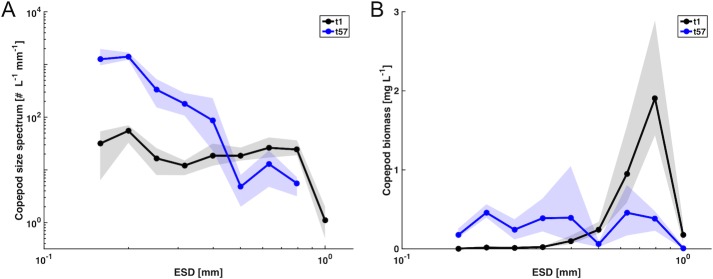
Size structure of the copepod community (including nauplii) in the mesocosms (average of 5 mesocosms in the control treatment). (A) normalized abundance spectrum and (B) weighted biomass spectrum. Shaded area denotes range of replicate mesocosms.

A striking feature in this regard is the pronounced change in size structure of the mesozooplankton community during the bloom (Fig 4). Before bloom onset, the community was dominated in terms of biomass by relatively large copepods (600 μm to >1 mm), with an only minor contribution of smaller specimen and nauplii. Throughout the bloom, abundance and biomass in the larger size class decreased substantially and copepods larger than 1 mm even disappeared completely.

In turn, a cohort of nauplii and small copepods (ranging between 200–600 μm) developed in response to the phytoplankton bloom and peaked around t57 (Fig 4). In the particle size and biomass spectrum, this can be perceived as a wave that propagates from small phytoplankton to copepods, and a simultaneous decrease in particle abundances and biomass in the size range between 600–1000 μm over the course of the bloom (Fig 2).

Altogether, these population dynamics resulted in a redistribution of copepod biomass from larger towards smaller body size (Fig 4). Notably, these shifts in size structure resulted in a decrease of overall copepod biomass by 25% between t1 and t57, despite the substantial increase in abundance in the smaller size classes (Fig 4, Table 1). Accordingly, the contribution of copepods to overall biomass of the system decreased considerably: While they constituted 63% of total biomass on t1, this number dropped to 18% on t57 (Fig 4, Table 1). In the biomass size spectrum, this becomes visible as a redistribution of biomass from larger (~600–1000 μm) to intermediate (200–600 μm) size classes, thereby diminishing the initially major contribution of large particles to overall biomass (Fig 2). It is noteworthy that despite these substantial changes in particle abundances in certain size classes, the overall slope of the particle size spectrum changed only marginally from -3.29 on t1 to -3.43 on t57 (R^2^ = 0.96).

### 3.2 Effects of ocean acidification on size spectra, biomass and food web structure

**Fig 5:**
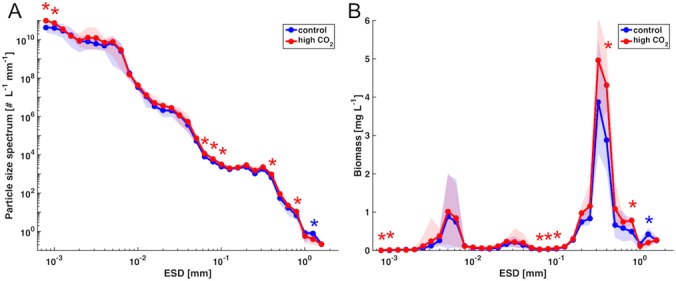
CO_2_ effects on particle size spectra. Comparison of normalized abundance spectrum (A) and weighted biomass spectrum (B) in the control (blue) and high CO_2_ mesocosms (red) on day t57. Shaded area denotes range of replicate mesocosms and asterisks indicate a statistically significant effect of CO_2_ on the respective size class (p<0.05).

**Fig 6:**
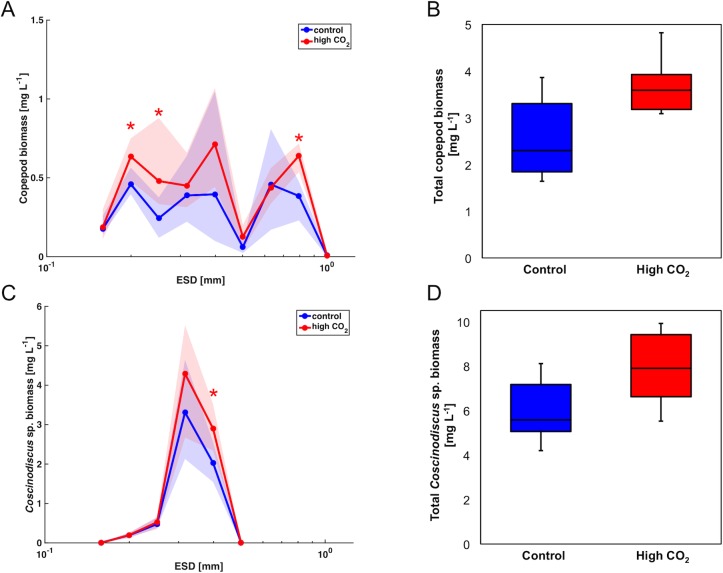
CO_2_ effects on the plankton community. CO_2_-related differences in biomass of copepods and nauplii (A,B) and *Coscinodiscus* sp. (C,D) in the control (blue) and high CO_2_ mesocosms (red) on day t57. Shown are the size distribution of biomass in weighted biomass-size spectra (A,C) and box plots for overall biomass (B,D). Shaded area denotes range of replicate mesocosms and asterisks in panel A and C indicate a statistically significant effect of CO_2_ on the respective size class (p < 0.05). Tests for statistical significance of total biomass in the respective groups (B,D) yielded p-values of p = 0.06 (copepods) and p = 0.10 (*Coscinodiscus*).

Simulated ocean acidification had a notable influence on the plankton community and food web structure during the bloom (peaking around t57). Particle abundances and biomass were elevated in response to high CO_2_ conditions over almost the entire size range (Fig 5). These CO_2_-related differences were statistically significant in distinct size classes that correspond to populations of different plankton groups, such as picoeukaryotes, *Coscinodiscus* sp., and parts of the copepod community (Figs 5 and 6).

A closer look at the size structure of the copepod community revealed statistically significant treatment effects (p<0.05) on copepod abundances and biomass in several size classes (~200–300 μm and 800–1000 μm ESD), mostly representing nauplii and small copepods, as well as some larger copepods (Fig 6A). Overall copepod biomass was elevated by 40% in the ocean acidification treatment compared to the control on average (Fig 6B, Table 2). However, despite the significant effect on several copepod size classes, this effect was just outside the level of significance for total copepod biomass (t-test, p = 0.06) due to substantial within-treatment variability of the control mesocosms. The size distribution of *Coscinodiscus* sp. revealed a statistically significant CO_2_ effect (p<0.05) on the largest size class (400–500 μm ESD) (Fig 6A). Accordingly, total biomass of *Coscinodiscus* sp. tended to be elevated by ~30% under high CO_2_ compared to the control mesocosms (Fig 6B, Table 2), although the effect was statistically not significant (t-test for t57, p = 0.10). In this regard, data from our plankton imaging approach are consistent with abundance data from manual countings that have been obtained independently (Bach et al., in prep.).

**Table 2:** CO_2_ effects on the plankton community. Average biomass of *Coscinodiscus* sp. and different size classes of copepods in the mesocosms under ambient conditions and under high CO_2_ on day t57. Shown is the average of five mesocosms (± standard error, SE).

Biomass in mesocosms on day t57 (treatment mean) [mm^3^ L^-1^]	Control			High CO_2_		
	Mean		SE	Mean		SE
*Coscinodiscus* sp.	6.02	±	0.67	7.92	±	0.78
Copepod total	2.58	±	0.40	3.68	±	0.31
Copepod *L* (> 1mm)	0.00	±	0.00	0.00	±	0.00
Copepod *M* (600–1000 μm)	0.84	±	0.13	1.08	±	0.05
Copepod *S* (<600 μm) & nauplii	1.73	±	0.32	2.60	±	0.31

**Fig 7:**
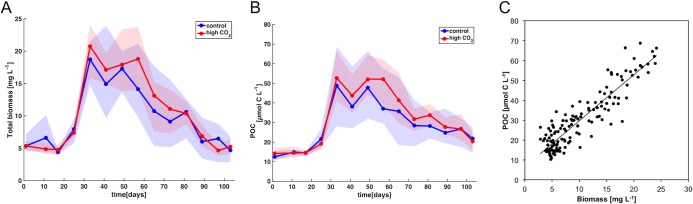
Evaluation of image-based method. Comparison of biomass development estimated from flow cytometry and Zooscan (A) with measurements of particulate organic carbon (POC) (B) in the control (blue) and high CO_2_ mesocosms (red). Shaded areas denote range of replicates. (C) Scatter plot comparing biomass estimated in this study with measured POC for all mesocosms and sampling days (n = 140, R^2^ = 0.83)

An opposite response to high CO_2_ was only visible in the size range >1 mm, which consisted almost exclusively of hydromedusae, but contributed only little to total biomass. As *Coscinodiscus* sp. and copepods accounted for ~50% and 20% of total biomass in the system, respectively, their size-specific responses to elevated CO_2_ were mirrored in an overall tendency towards elevated biomass in the ocean acidification treatment (Fig 6, Table 2). Total biomass was slightly elevated for an extended period during the plankton bloom (Fig 7A), even though this effect was statistically not significant due to the large variability within treatments (t-test for t57: p = 0.13).

### 3.3 Comparison of the plankton imaging approach with conventional measurements

All data on plankton abundance and biomass presented in this study were obtained by combining flow cytometry and a zooplankton imaging system. Generally, our data are in good accordance with other measurements that were conducted independently within the scope of the mesocosm campaign: Correlation coefficients for abundance data from microscopy and the image-based method were R^2^ = 0.93 for nauplii, R^2^ = 0.95 for copepods, and R^2^ = 0.96 for *Coscinodiscus* cells. Correspondingly, abundances and CO_2_-related trends of *Coscinodiscus* and copepods are consistent with manual counts that have been carried out independently (Bach et al., in prep., Algueró-Muñiz et al., in prep).

Furthermore, biomass estimates as determined from the image-based approach in this study agree very well with biogeochemical measurements of biomass (e.g. particulate organic carbon, POC, from a CN elemental analyzer [58]) that largely show the same temporal development and CO_2_-related trends (Fig 7B). Even the bloom magnitude is consistent between the two different approaches, both displaying a biomass increase by a factor of 3 to 4 from initial conditions to peak biomass (Fig 7B). Overall, there was strong correlation between estimated biomass and analytical measurements of POC (Fig 7C, R^2^ = 0.83), suggesting that our approach combining flow cytometry and zooplankton imaging is well suitable for estimating biomass and productivity of entire plankton communities.

## 4 Discussion

The plankton imaging approach applied in our study revealed distinct temporal shifts in the size structure of the mesozooplankton community over the course of a plankton spring bloom. Remarkably, our findings suggest a positive effect of elevated CO_2_ on copepod biomass during the plankton succession. In the following, we discuss the most likely mechanisms behind these observations, as well as their implications for food-web structure and ecosystem functioning during the mesocosm experiment.

### 4.1 Effects of ocean acidification on the plankton community

Simulated ocean acidification had an effect on both phytoplankton and zooplankton, even though the magnitude of this effect was very variable among different plankton groups, and only occurred during certain stages of the succession (Bach et al., in prep.). The mesozooplankton community in the mesocosms was heavily dominated by the calanoid copepod *Pseudocalanus acuspes*. Interestingly, copepod biomass was elevated under high CO_2_ conditions, with biomass (in distinct size classes) being 40% higher than in the control mesocosms on day t57 (Fig 6, Table 2). However, it seems unlikely that elevated CO_2_ concentrations exerted a direct positive effect on copepods. The majority of studies so far have found copepods to be rather resilient to increasing CO_2_ [14, 15, 74], or indicate negative effects of ocean acidification on earlier developmental stages of copepods [17, 18]. For *Pseudocalanus acuspes*, previous evidence suggests rather negative physiological effects of OA, e.g. by increasing respiration, and decreasing ingestion and fecundity [75, 76]. Regarding the positive effect of elevated CO_2_ on copepods in our experiment, the most likely explanation seems to be that there was a bottom-up effect at play.

Elevated pCO_2_ had a stimulating effect of on phytoplankton in our mesocosm study, resulting in higher primary production and biomass of phytoplankton (Eberlein et al., submitted, Bach et al., in prep.). Our observations suggest that, this increase in phytoplankton productivity propagated up the food web and ultimately became visible as elevated biomass of copepods. In a broader sense, these findings imply that increased food availability in the OA treatment (indirect effect) compensated for a potentially negative impact of high CO_2_ on physiology of copepods (direct effect). These considerations are consistent with observations from previous laboratory studies, which indicated that physiological performance of copepods is influenced much stronger by food availability than by direct effects of elevated CO_2_ [75]. Therefore, our study demonstrates for the first time in natural plankton communities how effects of elevated CO_2_ on primary producers can translate into indirect OA responses of important secondary producers, such as copepods.

As a consequence, the question arises where exactly this CO_2_-related food web effect originates from. The phytoplankton community consisted mostly of picoeukaryotes, some nanophytoplankton groups (mainly small diatoms), and the very large diatom *Coscinodiscus* sp. (see Bach et al., in prep.). In terms of food sources for mesozooplankton, it is unlikely that *Coscinodiscus* sp. was available as prey for the copepod community in the mesocosms due to their large cell size of 200 to >400 μm ESD (Figs 1 and 6). Since the largest individuals of the dominant copepod *Pseudocalanus acuspes* were in the range of 600–1000 μm ESD, *Coscinodiscus* cells were far out of a typical predator-prey size ratio in the range of 10:1 to 30:1, which would be a reasonable range estimate for most filter-feeding copepods [35]. Accordingly, the indirect CO_2_ effect on copepods must largely originate from a different part of the food web. It is likely that biomass produced by nanophytoplankton in the size range of 2–15 μm was the main food supply for the copepod community and largely sustained the population increase of nauplii and copepods. However, no consistent effects of elevated CO_2_ could be detected for the different nanophytoplankton groups, thus not providing a straightforward explanation for the observed CO_2_ effect on copepod biomass (Bach et al., in prep.). Nevertheless, based on observed CO_2_-related differences in rates of primary production (Eberlein et al., submitted), it is reasonable to assume that there was a larger surplus in nanophytoplankton production under high CO_2_ that was directly grazed upon, thus not becoming visible in standing stocks.

Another possibility is that the CO_2_ effect on copepods was the result of more complex food-web interactions, including an additional trophic level. The most prominent CO_2_ effect on phytoplankton occurred in the size range of picoeukaryotes, which displayed notably higher abundances in the ocean acidification treatment during the second bloom (>60% higher, Fig 5A; also see Bach et al., in prep.). However, considering their small size (< 2 μm), it seems unlikely that picophytoplankton were directly grazed upon by developing *Pseudocalanus* nauplii or copepodites, which usually rather feed in the nano- and microplankton size range [77]. Potentially, increased growth rates and abundances of picoeukaryotes resulted in enhanced grazing by microzooplankton such as ciliates (see Horn et al., accepted), which in turn could have provided an additional food source for copepods. Although *Pseudocalanus* have been found to be predominantly herbivorous [77–79], some studies shown that they also graze on microzooplankton or detritus when phytoplankton biomass is insufficient to sustain their food demand [80, 81]. Interestingly, ciliate abundances display no or a rather negative response to high CO_2_ conditions (Horn et al., accepted). Nevertheless, it is possible that the beneficial effect of increased prey abundance (picoeukaryotes) on ciliates under high CO_2_ was masked by immediately transferring this extra biomass of microzooplankton one trophic level further up the food web, i.e. due to strong grazing pressure by copepods, as well as by fish larvae during this time of the experiment (see Horn et al., accepted, Sswat et al., in prep.). Such a trophic cascade could explain why CO_2_ effects become visible in certain parts of the food web (here copepods), but not in others.

Clearly, the response of copepods to ocean acidification depends not only on changes in food quantity, but also on how food quality might be affected by elevated CO_2_. Most laboratory-based studies so far found rather negative effects of deteriorating food quality at higher CO_2_ on copepods, e.g. due to unfavorable biochemical composition of phytoplankton prey [82–84]. While we do not have direct information about potential changes in food quality in our experiment, the positive response of copepods to elevated CO_2_ indicates that the CO_2_-driven increase in food quantity (due to enhanced primary productivity and phytoplankton biomass) outweighed potentially negative effects on food quality that might have occurred under high CO_2_ conditions.

In summary it can be stated that simulated ocean acidification had a profound impact on the plankton community and food web structure, which ultimately propagated up the food web and resulted in enhanced biomass of the copepod community. Ultimately, there is no simple and obvious explanation where the distinct effect of elevated CO_2_ on the copepod community originated from. It is likely that the observed CO_2_ effect on abundance and biomass of copepods was the result of complex food-web interactions, possibly also driven by production of biomass that is not visible from standing stock concentrations. From the primary producers’ perspective, this would imply that stimulating effects of CO_2_ on build-up of phytoplankton biomass (Eberlein et al., submitted, Bach et al., in prep.) were potentially obscured by immediate transfer of this extra biomass up the food web.

It is noteworthy that the positive CO_2_ effect on the copepod community even reached the next higher trophic level, which is indicated by the increased survival of herring larvae in the mesocosms with elevated CO_2_ conditions (Sswat et al., in prep.). Altogether, these findings highlight the importance of indirect CO_2_ effects and consequential trophic cascades for assessing the responses of food web structure and ecosystems to ocean acidification.

### 4.2 What shaped the size structure of the food web?

Besides effects of elevated CO_2_, our plankton imaging approach yielded interesting insights into temporal changes in the size structure of the copepod community, with a pronounced shift towards smaller sizes and an associated decrease in overall copepod biomass irrespective of the CO_2_ treatment (Fig 4). These observations raise the question for the reasons behind this shift in size structure. Initially, top-down control by large copepods (600–1000 μm) on microphytoplankton could have suppressed a phytoplankton bloom in this size range, thereby giving rise to the observed blooms of nanophytoplankton and large *Coscinodiscus* sp., which then consumed a major portion of inorganic nutrients. In other words, size-selective grazing pressure likely cut out a confined part of the size spectrum (~30–200 μm), which subsequently gave rise to the distinct bi-modal size distribution of the phytoplankton bloom (Fig 2) (Bach et al., in prep.). The biomass produced by the nanophytoplankton bloom (~2–15 μm) was in a suitable size range for grazing by copepod nauplii and small copepods, and thus likely their primary food source. For larger adult copepods, however, this prey size range was potentially too small for efficient feeding or contained too little biomass to sufficiently nourish them. This finding would be in line with previous studies, which suggest that juvenile copepods reach half-saturation of growth at food concentrations an order of magnitude lower than adults [31].

As mentioned above, there is evidence that *Pseudocalanus* can switch from a predominantly herbivorous feeding mode [77], to omnivorous grazing when its preferred prey is scarce [80, 81]. In the case of our experiment, this would encompass prey in the size range of approximately 50–100 μm, which mostly comprised ciliates and other microzooplankton. However, since particle abundances in this size range were rather low until around t40 (Fig 2A and 2B), microzooplankton could likely not provide a sufficiently large alternative food source to sustain the demand of larger copepods in the mesocosms. Altogether, the initial top-down control of microphytoplankton by larger copepods likely prevented the former from blooming, which in turn resulted in a lack of food in the required size class for large adult copepods early in the experiment and might ultimately be the main reason for their decline and the complete disappearance of large copepods by day t57 (Fig 4).

Yet, it is somewhat surprising that the developing cohorts of nauplii and small copepods that were present in high abundance during the time of peak biomass (t57) did not translate into higher biomass at larger sizes later in the experiment (Fig 2B). Usually, one would expect a propagation of zooplankton cohorts along the size spectrum, i.e. the development of a larger-sized cohort of adult copepods some time during or after the bloom. This was, however, not observed in our study, where biomass in the size range of ~800 μm to 1 mm (corresponding to adult *Pseudocalanus acuspes*) rather decreased throughout the study (Figs 1 and 4, also see Algueró-Muñiz et al., in prep). Again, one reason might be the lack of sufficient prey biomass in a size range that is available to larger copepods. Furthermore, copepodites and nauplii might have experienced insufficient food supply later in the experiment (starting around day t80), when nanoplankton biomass decreased severely in the post-bloom phase and potentially led to starvation of developing copepods (Fig 2B). Another important factor might have been predation by herring larvae (*Clupea harengus)*. Herring eggs were introduced into the mesocosms on day t48, with peak hatching occurring on day t63 ([58], Sswat et al, in prep.). Newly hatched herring larvae most likely started feeding on small particles in the size range of 50–200 μm (e.g. ciliates and copepod nauplii), switching to bigger prey such as copepodites with growing body size. Thus, predation of herring larvae on different developmental stages of copepods might be an additional reason why the high abundances of nauplii and copepodites did not translate into an increase of larger, adult copepods in the second half of the experiment. It might also be possible that at least part of the adult copepods began migrating vertically in the water column and thereby got lost in the sediment trap at 19 m depth. Evidence for this behavior was obtained in a previous mesocosm study in a Norwegian fjord, where relatively high numbers of copepods were found in the sediment traps, particularly at times of low food availability [85]. Unfortunately, we were not able to quantify this potential loss in the present study, but we are aware that vertical migration of adult copepods and associated loss in the sediment trap might have contributed to the low presence of large copepods, particularly towards the end of the experiment when food availability was declining.

Altogether, it was probably the interplay of all above-mentioned mechanisms that ultimately resulted in the observed shifts in size structure of the copepod community and the associated decline of overall mesozooplankton biomass during the study. These observations highlight the importance of monitoring changes in plankton size distribution for investigations of food web structure and ecosystem functioning.

### 4.3 Particle size spectra: Observations vs. theory

Interestingly, the particle size spectra observed in this study did not closely match a power-law distribution as expected from theory [42, 46, 86]. Instead of following a quasi-linear relationship on a log-log plot, particle size spectra in this study rather displayed the development of distinct waves in certain size classes (e.g. Figs 2C and 5A). However, it should be noted that even though the power-law distribution in size spectra is well-documented and forms the basis of several empirical and theoretical models, most of the existing studies that reported a power-law pattern averaged data over quite large spatial and / or temporal scales [44, 87]. When considering the seasonality and spatial heterogeneity of many marine ecosystems, it becomes clear that there must be strong deviations from the power-law steady state, e.g. through phytoplankton blooms or different developmental stages of zooplankton, as could be observed in our mesocosm experiment. In fact, studies with more frequent sampling and data over a longer time period report substantial variability of size spectra throughout the year [41, 43, 48], which would not be visible when aggregating size spectra over longer temporal scales. Thus, the typical power-law”steady state” of marine particle size spectra may not be found at any specific time, but rather represents an average state over large timescales.

Another interesting result was that changes in the slope of the particle size spectrum were marginal in our experiment, although we observed a pronounced plankton bloom and substantial temporal variations in biomass in specific size classes. Based on recent work in size spectrum theory, the slope of the normalized abundance spectrum is often used as an indicator for the trophic structure of the system: Steeper slopes are often assumed to be indicative for a high degree of herbivory and efficient biomass transfer up the food web with a relatively low number of trophic levels, while flat slopes are associated with a higher number of trophic levels, i.e. systems dominated by recycling and carnivory or detrivory [42, 47]. Furthermore, the slope of the size spectrum has been used to compute rates of secondary production and mortality of mesozooplankton in such studies. With regard to our experiment, the almost negligible change in slope of the size spectrum despite major changes in biomass and food web structure, make it seem questionable if these concepts are applicable for highly dynamic plankton food webs establishing and developing during plankton successions. As visible from our data (Fig 2A and 2C), a linear fit does not adequately describe the observed changes in the size spectrum that occurred during the bloom, which exhibited several distinct “waves”, each representing the temporal dynamics of different plankton groups (e.g. nanophytoplankton, nauplii, copepods, *Coscinodiscus*). Furthermore, the slope of the size spectrum is strongly determined by the abundance of grazers of larger size. However, in the case of the mesocosm experiment presented here, the size overlap of smaller copepods and the large diatom *Coscinodiscus* (both between ~200–500 μm ESD, see Figs 1 and 3) make it impracticable to simply use the slope of the size spectrum as an indicator for food web structure.

### 4.4 Applicability of plankton imaging for ecosystem studies: Comparison with conventional methods, caveats and potential future improvements

In this study, we used flow cytometry and a plankton imaging system to acquire data on abundance, size and biomass of particles and organisms. By combining the two instruments, we obtained size-continuous data of particles in the water column, spanning several orders of magnitude in size (from ~0.5 μm to more than 1 mm).

Our plankton imaging approach revealed pronounced shifts in size structure of the copepod community, resulting in a substantial decrease in overall copepod biomass (see section 3.1 and 4.2, Fig 4). Interestingly, these developments are not visible in data of mesozooplankton abundance counted with a stereomicroscope (Algueró-Muñiz et al., in prep), which do not account for shifts in size distribution: Abundance estimates from microscopy show little change of adult copepods throughout the experiment, but much higher abundances of nauplii and copepodites during the bloom. Such an overall increase in copepod abundance would usually be interpreted as an increase of copepod biomass during the bloom. However, our image-based approach suggests that the opposite occurred and the pronounced shift in copepod size distribution even resulted in a marked decrease of overall copepod biomass during the bloom. It should be emphasized that these findings do not imply a discrepancy between microscopy data and our plankton imaging approach, but rather demonstrate how additional information from image-based methods (such as size structure) can shed more light on population dynamics and food web structure.

One issue that should be noted is that the flow cytometry (FCM) measurement was triggered on fluorescence, as the primary focus of this method was to investigate phytoplankton community dynamics. Although already very low levels of fluorescence are sufficient to trigger the FCM, it is possible that we missed some (non-fluorescing) particles in the size range covered by this instrument (~0.5 to 60 μm ESD). However, parallel measurements with an imaging flow cytometer (Cytosense) revealed that many non-phytoplankton particles were actually detected by the instrument. For instance, practically all microzooplankton cells contained at least some fluorescing pigments in their guts, thereby making them detectable by the FCM. Therefore, we are confident that the vast majority of particles in the microplankton size range (>20 μm) were adequately represented in our data. It should be noted, however, that smaller non-fluorescing particles (e.g. bacteria, viruses, small detritus) were not detected by the flow cytometer (see methods section). Although particle counts in these size ranges are usually heavily dominated by phytoplankton cells, we cannot exclude that such small non-fluorescing particles might have been present and contributed to total abundances and biomass to some extent. Therefore, it is advisable to set flow cytometers on forward (or sideward) scatter triggering to avoid any bias towards fluorescent particles for future studies following this approach.

Despite these methodological issues, our data from the image-based method was overall in very good agreement with other datasets that were obtained independently, e.g. manually counted abundances of copepods and *Coscinodiscus* sp., as well as measurements of particulate organic carbon concentrations (see section 3.3 and Fig 7C). Thus, we are confident that our approach of combining flow cytometry with plankton imaging for a holistic description of plankton community structure can be a valuable tool for future studies of community composition, food web structure and estimates of biomass production of marine ecosystems.

## 5 Conclusion

In this study we combined flow cytometry and an image-based approach to follow the development of a natural plankton community (ranging from picoplankton to mesozooplankton) over the course of a spring bloom and winter-to-summer succession, with a particular focus on potential effects of ocean acidification. Our methodological approach proved as a powerful tool for investigating size distribution, community composition and food web structure of marine plankton communities, yielding interesting insights into the coupling of primary producers and top-down control by zooplankton grazers.

Our observations revealed distinct shifts in the size structure of the copepod community towards smaller body size, leading to a substantial decrease of mesozooplankton biomass over the course of the plankton bloom.

Moreover, we observed a pronounced positive effect of elevated CO_2_ concentrations as expected for the end of the century (~760 μatm *p*CO_2_) on size structure and biomass of the copepod community (dominated by *Pseudocalanus acuspes*). Our findings suggest that ocean acidification can have (indirect) positive effects on important secondary producers such as copepods, when CO_2_-enhanced primary production and biomass of phytoplankton are transferred up the food web. Since copepods serve as a major food source for a variety of commercially important fish species, such CO_2_-driven trophic cascades could have important implications for ecosystem structure and fish stock dynamics in temperate and arctic regions.
